# Enhanced Corticospinal Excitability and Volitional Drive in Response to Shortening and Lengthening Strength Training and Changes Following Detraining

**DOI:** 10.3389/fphys.2017.00057

**Published:** 2017-02-07

**Authors:** Jamie Tallent, Stuart Goodall, Karl C. Gibbon, Tibor Hortobágyi, Glyn Howatson

**Affiliations:** ^1^Department of Sport, Exercise and Rehabilitation, Northumbria UniversityNewcastle-upon-Tyne, UK; ^2^School of Sport, Health and Applied Science, St Mary's UniversityTwickenham, UK; ^3^Department of Advanced Health Science, Buckinghamshire New UniversityHigh Wycombe, UK; ^4^Faculty of Medical Sciences, University of GroningenGroningen, Netherlands; ^5^Water Research Group, School of Environmental Sciences and Development, Northwest UniversityPotchefstroom, South Africa

**Keywords:** concentric, eccentric, PNS, resistance, TMS, exercise

## Abstract

There is a limited understanding of the neurological adaptations responsible for changes in strength following shortening and lengthening resistance training and subsequent detraining. The aim of the study was to investigate differences in corticospinal and spinal responses to resistance training of the tibialis anterior muscle between shortening or lengthening muscle contractions for 4 weeks and after 2 weeks of detraining. Thirty-one untrained individuals were assigned to either shortening or lengthening isokinetic resistance training (4 weeks, 3 days/weeks) or a non-training control group. Transcranial magnetic stimulation and peripheral nerve stimulation (PNS) were used to assess corticospinal and spinal changes, respectively, at pre-, mid-, post-resistance training and post detraining. Greater increases changes (*P* < 0.01) in MVC were found from the respective muscle contraction training. Motor evoked potentials (expressed relative to background EMG) significantly increased in lengthening resistance training group under contraction intensities ranging from 25 to 80% of the shortening and lengthening contraction intensity (*P* < 0.01). In the shortening resistance training group increases were only seen at 50 and 80% of both contraction type. Volitional drive (V-wave) showed a greater increase following lengthening resistance training (57%) during maximal lengthening contractions compared to maximal shortening contractions following shortening resistance training (23%; *P* < 0.001). During the detraining period MVC and V-wave did not change (*P* > 0.05), although MEP amplitude decreased during the detraining period (*P* < 0.01). No changes in H-reflex were found pre to post resistance training or post detraining. Modulation in V-wave appeared to be contraction specific, whereby greatest increases occurred following lengthening resistance training. Strength and volitional drive is maintained following 2 weeks detraining, however corticospinal excitability appears to decrease when the training stimulus is withdrawn.

## Introduction

Increases in muscle strength through resistance exercise are essential for both clinical and athletic populations and consist of shortening and lengthening contractions that possess their own unique properties. Lengthening contractions can increase lengthening strength three-fold and neural output (EMG) seven-fold more compared to shortening contractions following shortening resistance training (Hortobágyi et al., 1996). In a recent meta-analysis (Roig et al., 2009), it was shown that lengthening contractions are more effective at increasing strength and muscle mass, though the neurological adaptations were not discussed. Despite many studies supporting the notion that lengthening contractions are superior to shortening contractions not all research concurs (Higbie et al., 1996; Andersen et al., 2005). Lengthening contractions have generally been shown to have a positive effect on clinical populations such as stroke patients (Engardt et al., 1995). In athletes, increases in lengthening contraction strength are associated with a reduction in injury prevalence (Jönhagen et al., 1994). Surface EMG, a surrogate measure of neural drive detected at the muscle, has shown muscle activity to be reduced following the cessation of resistance training (Häkkinen and Komi, 1983; Gondin et al., 2006); however, it is not clear if these changes in neural drive are attributable to supraspinal and/or spinal mechanisms. Investigating the unique control strategies (Duchateau and Enoka, 2008; Duclay et al., 2011) of shortening and lengthening contractions and the neural modifications from resistance training and detraining will aid clinicians and practitioners in training and rehabilitation programme design. Understanding where the adaptations (cortical, spinal or post-synaptic) occur for each muscle contraction will assist practitioners in maximizing neurological and consequently, strength adaptations.

To detect and monitor changes in the central nervous system (CNS) from resistance training, transcranial magnetic stimulation (TMS) and peripheral nerve stimulation (PNS) have been used to identify the possible mechanisms of adaptation (Kidgell et al., 2010; Tallent et al., 2013). However, despite the large body of research focusing on the early neurological adaptations to resistance training, little continuity exists in the literature. An increase (Griffin and Cafarelli, 2007; Kidgell et al., 2010), decrease (Carroll et al., 2002), or no change (Carroll et al., 2009) in corticospinal excitability and reduced (Kidgell and Pearce, 2010) or no change in inhibition (Kidgell et al., 2010; Weier et al., 2012) have been reported following a period of resistance training. At a spinal level, an increased excitability has been reported in some studies (Aagaard et al., 2002; Gondin et al., 2006), but not others (Holtermann et al., 2007; Ekblom, 2010). However, V-wave has consistently been shown to increase (Aagaard et al., 2002; Gondin et al., 2006; Ekblom, 2010; Vila-Chã et al., 2012). Due to reasons such as reproducibility, research has focused predominantly on assessment during isometric contractions.

Lengthening contractions show a greater neuromuscular response to training than shortening contractions. It has been shown that shortening muscle contractions coupled with lengthening contractions have a greater preservation of strength, which is potentially due to the greater neurological adaptations from lengthening contractions (Colliander and Tesch, 1992). Conversely, adaptations following lengthening training have also been shown to be less susceptible to detraining (Andersen et al., 2005). The neurological mechanisms behind these preservations are unclear. A greater understanding of the contributing factors to the decreases in strength in response to detraining has important applications for the design of taper strategies, but more importantly furthers our understanding of how detraining or inactivity affects neuromuscular function (Bosquet et al., 2013). The collective use of tools, such as TMS and PNS, to explore the nervous system during shortening and lengthening contraction could further our knowledge of the responses to resistance training and detraining following task specific shortening or lengthening contractions. With little understanding of the nervous system following detraining, TMS and PNS may also further our understanding of periods of inactivity.

The study was split into two parts/aims. The initial aim of this study was to investigate the response to 4 weeks shortening or lengthening resistance training; secondly, we aimed to examine the detraining response following 2 weeks of no training. TMS and PNS was employed to investigate corticospinal and spinal task-specific changes following training and detraining. It was hypothesized that corticospinal and spinal adaptations following resistance training would be modulated in a contraction-specific manner. It is also suggested that such changes would be greater following lengthening resistance training because of the potential greater training responses observed following this mode of exercise.

## Materials and methods

### Subjects

Following institutional ethical approval from the University of Northumbria Ethics Committee and in accordance with the Declaration of Helsinki, 31 male volunteers completed a health-screening questionnaire and provided written, informed consent. Participants had no structured resistance training history in the preceding 2 years and were randomly assigned to either a shortening resistance training (SHO; *n* = 11), lengthening resistance training (LEN; *n* = 11), or a control (CON; *n* = 9) group. Mean ± *SD* age, stature and mass were 24 ± 3, 24 ± 3 and 27 ± 4 yrs, 175.9 ± 10.6, 176.3 ± 9.6, 172.7 ± 8.5 cm, and 77.1 ± 10.2, 75.7 ± 12.3, 74.7 ± 11.1 kg, respectively). Participants were asked to refrain from any form of resistance training throughout the duration of the study. Footedness was assessed using a questionnaire (Hebbal and Mysorekar, 2006); of the 31 participants, 29 were right leg dominant.

### Study design

The training groups conducted an initial 4 weeks of resistance training and then 2 weeks detraining, with the CON group remaining inactive throughout the 6 week period. Participants allocated to the resistance training group reported to the laboratory on 17 occasions; 5 times for assessments and 12 times for training sessions. The CON group took part in the five assessments only. All participants performed a familiarization session 24 h before the pre-testing assessment (Tallent et al., 2013). Midpoint assessment was conducted after 2 weeks (six training sessions) and post-training assessment was after 4 weeks (12 training sessions). Final measures were taken after 2 weeks of detraining (weeks 6) where participants were instructed to remain inactive during this time. Preliminary power analysis using GraphPad StatMate (v5.0, San Diego, CA, USA), and previous work (Hortobágyi et al., 1997, 2000), revealed that to achieve a power of 0.80 (α = 0.05), 10 participants per experimental group were needed. However, as no participants withdrew from the study, the experimental groups consisted of 11 subjects in each individual experimental training group with nine in the control group.

### Resistance training

All assessment and training sessions were conducted on an isokinetic dynamometer (Cybex Norm, Cybex International, NY). The experimental groups performed 12 sessions of LEN or SHO resistance training of the tibialis anterior (TA) muscle. The TA was used due to its uniquely high corticospinal drive (Capaday et al., 1999) and accessibility of the common peroneal nerve through electrical stimulation. Training consisted of 3 sessions per week. Session 1–5 and 7–11 were conducted at 80% of the relative contraction specific MVC. Sessions 6 and 12 were reduced to 50% MVC to minimize any potential fatigue during the assessment sessions. Following a warm-up set of 8 reps at 50% MVC, participants performed 5 sets of 6 repetitions at 80% MVC of contraction specific MVC at a speed of 15°/s with 2 min rest between sets. Supervised training was conducted under identical conditions to the assessment sessions apart from a shorter 2 s rest period between each repetition. All participants performed all training sessions, with training sessions taking maximum of 20 min. MVC was recorded during each assessment session and training torque was adjusted where necessary at the mid assessment point.

### Experimental set-up

Participants were set-up on the isokinetic dynamometer in accordance with manufacturer's guidelines to examine the dorsiflexor muscle group (hip, knee and ankle of the dominant leg set at joint angles of 90, 120, and 90°, respectively). The knee and foot was strapped into avoid any unwanted movements in the leg. Whilst performing contractions participants were instructed not to grip the handles of the dynamometer. Torque feedback was displayed on the dynamometer's monitor which was set 1 m away from the participant. Participants moved the ankle joint through a 30° range (75°–105°, where 90° was anatomical zero) of dorsi- and plantar-flexion at a speed of 15°/s, resisting for lengthening contractions and assisting for shortening contractions. Following MVC assessments in each laboratory visit, TMS and PNS was performed in a randomized order.

### Maximal voluntary contraction (MVC)

After a warm-up, consisting of shortening and lengthening (SHO and LEN) contractions at 60, 80, and 90% of maximal effort, three SHO and LEN maximal voluntary contractions (MVC) were performed. Participants had three MVC attempts with 2 min rest between each contraction. If the third MVC attempt recorded the highest value a fourth MVC was performed. The highest torque as the ankle passed 90° for each individual MVC was recorded. From these values, 80, 50, 25, and 15% of shortening and lengthening MVC were calculated.

### TMS protocol

The repeatability of the TMS and PNS measures used in this study have been previously established during both shortening and lengthening contractions performed with the TA (Tallent et al., 2013). Consequently, the same protocol was used to assess corticospinal and spinal changes resulting from the training intervention. MEPs were elicited via stimulation on the hemisphere contralateral to the dominant leg using a magnetic stimulator (Magstim 200^2^, Magstim Company Ltd, Whitland, UK), with a concave double-coned 110 mm coil (maximal output of ~1.4 T). EMG was recorded in a 550 ms window (50 ms before magnetic stimulation and 500 ms after). Positioning for the optimal coil placement began 1 cm posterior to the vertex, along the sagittal plane. The coil was set-up to induce a postero-anterior current in the underlying motor cortex. The optimal site was marked with a permanent marker to ensure consistency in coil placement across the trial. Resting motor threshold (rMT) was defined as the lowest stimulator output to elicit a peak-to-peak MEP amplitude (≥50 μV) in 5 out of 10 consecutive pulses (Rossini et al., 1994). All subsequent pulses in the experiments were delivered at 120% of rMT. All TMS pulses were delivered when the ankle joint angle was at 90° (anatomical zero) and the resultant MEP amplitudes were normalized to peak-to-peak M_MAX_ amplitude. The order of contraction (lengthening and shortening) and intensity (80, 50, 25, 15% MVC) were randomized and counterbalanced. MEP's were examined at a range of contraction intensities to investigate the notion that amplitude changes could be more prominent at task specific intensities. Furthermore, the order of TMS and PNS was also randomized.

### Peripheral electrical stimulation

Electrical stimulation (pulse width, 1 ms) was administrated below the head of the fibula, over the peroneal nerve using a 40 mm diameter cathode/anode arrangement (Digitimer DS7AH, Welwyn Garden City, Hertfordshire, UK). We have previous shown both H-reflex and V-wave can be reliably performed in the TA (Tallent et al., 2012). A 10–15% isometric MVC was used to stabilize the H-reflex for resting conditions. After the optimal position for the stimulator was established, it was secured in place. The position was marked with semi-permanent ink to ensure consistency in placement across the trials. The site was checked at the beginning of each assessment session to ensure it was still the optimal site for stimulation. Up to 64 pulses from the first appearance of the H-reflex to M_MAX_ were delivered; the H-reflex at rest was defined as the average of the three highest responses. At least 25 s was left between pulses to ensure the H-reflex has return to pre resting values (Howatson et al., 2011).

After establishing the highest H-reflex, participants performed 12 shortening and 12 lengthening contractions at 25% of MVC, with 60 s between contractions. Stimulator output was adjusted to elicit an M-wave amplitude of 15–25% of M_MAX_. H-reflex was expressed relative to the contraction type and intensity. In the same manner as MEP's, H-reflex was also expressed relative to background EMG. Participants were passively moved in to position 10 s before performing a submaximal contraction and performed a 10–15% MVC to prevent any thixotropic effect. V-waves were examined under four shortening and lengthening contractions. A supramaximal stimulus intensity (150% of M_MAX_) was used to induce V-wave during the maximal contraction (Aagaard et al., 2002).

### Surface electromyography (EMG)

EMG sites were shaved, abraded with preparation gel and then wiped clean with an alcohol swab. Surface EMG was recorded over the TA using pairs of electrodes (20 mm diameter, model; Kendall, Tyco Healthcare Group, Mansfield, MA, USA) spaced 2 cm apart. For the TA, electrodes were placed at one-third distance of the line between the tip of the fibula and the tip of the medial malleolus (Hermens et al., 2000). The reference electrode was placed over the medial malleolus. EMG was amplified (× 1000), band pass filtered 10–1000 Hz (D360, Digitimer, Hertfordshire, UK) and sampled at 5000 Hz (CED Power 1401, Cambridge Electronics Design, Cambridge, UK). Despite the well-documented potential limitations of EMG (Farina et al., 2004), particularly during dynamic contractions where skin movement across the muscle is increased, our laboratory has previously demonstrated that this method is repeatable (Tallent et al., 2012), which is probably attributable to the fact the joint angle is identical between conditions when the stimulation is delivered and therefore delimits the potential for this to be a confounding factor.

### EMG processing

All TMS responses were averaged from eight responses and all contractions were separated by 30 s. The time from the stimulation artifact to the return of pre-stimulus EMG (within 1 SD) was determined as the silent period length. MEP's were also expressed relative to background EMG activity. EMG was rectified with the mean muscle activity 25 ms following the stimulator artifact and the average amplitude was recorded.

### Ultrasound

To detect any change in muscle thickness, a real-time digital ultrasound imager (Technos MP, Esaote, Genoa, Italy) in B-mode was used to collect sonographic images of the resting TA at the beginning of each experimental test session. A 40 mm linear-array transducer (CA621, Esaote, Genoa, Italy) with a variable center frequency (5–13 MHz) was placed over the longitudinal axis of the TA at a standardized position for all participants. The optimal position was 20% of the distance between the head of the fibula to the lateral tip of the lateral malleolus (Martinson and Stokes, 1991). The transducer head was placed perpendicular to the skin along the palpable edge of the tibia and adjusted obliquely to optimize visualization of echogenic landmarks, specifically the deep edge of the tibia, muscle fascicles and the central aponeurosis. Water-soluble hypoallergenic ultrasonic transmission gel (Aquasonic 100, Parker Laboratories Inc., Fairfield, New Jersey) was applied to the head of the transducer probe prior to skin placement. All images were taken unilaterally on the dominant limb with the participant lying supine with their ankle held in a neutral position. Images were captured in triplicate and exported for later analysis offline using publicly available software (Image J, US National Institutes of Health, available at http://rsb.info.nih.gov/ij/). Linear muscle thickness (LMTh), previously shown to reflect cross-sectional area (Martinson and Stokes, 1991), was determined as the distance between the inferior boundaries of the echogenic muscle fascia.

### Statistical analyses

All data were screened for normal distribution. Not normally distributed data was log transformed. To ensure resistance training was conducted at the same relative contraction intensity between groups, a time (training session 1–12) × group (SHO, LEN, CON) repeated measure ANOVA was performed. To confirm TMS and PNS variables were assessed under the same relative torque conditions between groups (SHO, LEN, CON) and across time (PRE, MID, POST) a repeated measured ANOVA was performed.

Percentage change in MVC and the corticospinal silent period, H-reflex and V-wave were examined by a repeated measures ANOVA (time [PRE, MID, POST] × group [SHO, LEN, CON] × contraction type [shortening, lengthening]). Finally, differences in MEP amplitude were assessed using repeated measures ANOVA: time × group × contraction type × contraction intensity (15, 25, 50, 80% of MVC). Additionally, 95% confidence intervals (CI) were determined to assess the magnitude of change. Statistical analyses were performed using SPSS (Chicago, Illinois, USA). To detect changes in dependent measures from the training and detraining periods, repeated measures ANOVA's were performed. Where necessary, bonferroni *post-hoc* tests were performed for pairwise comparisons. Significance was accepted as ≤ 0.05.

## Results

### Training and assessment

All data is presented as mean ± *SD*. For the raw data relative to M_MAX_ please see Supplementary Tables 1–3. There was no difference (*P* > 0.05) in relative training intensity between the SHO and LEN training groups, for the 12 resistance training sessions. The repeated measured ANOVA showed no differences (*P* > 0.05) in relative torque during the assessment sessions across time between groups and relative contraction intensity. Similarly, there was no significant difference in torques during PNS (*P* > 0.05) assessment sessions. There were no changes in muscle thickness across the training period (*P* = 0.76).

### Maximal voluntary contraction

**Figure 1:**
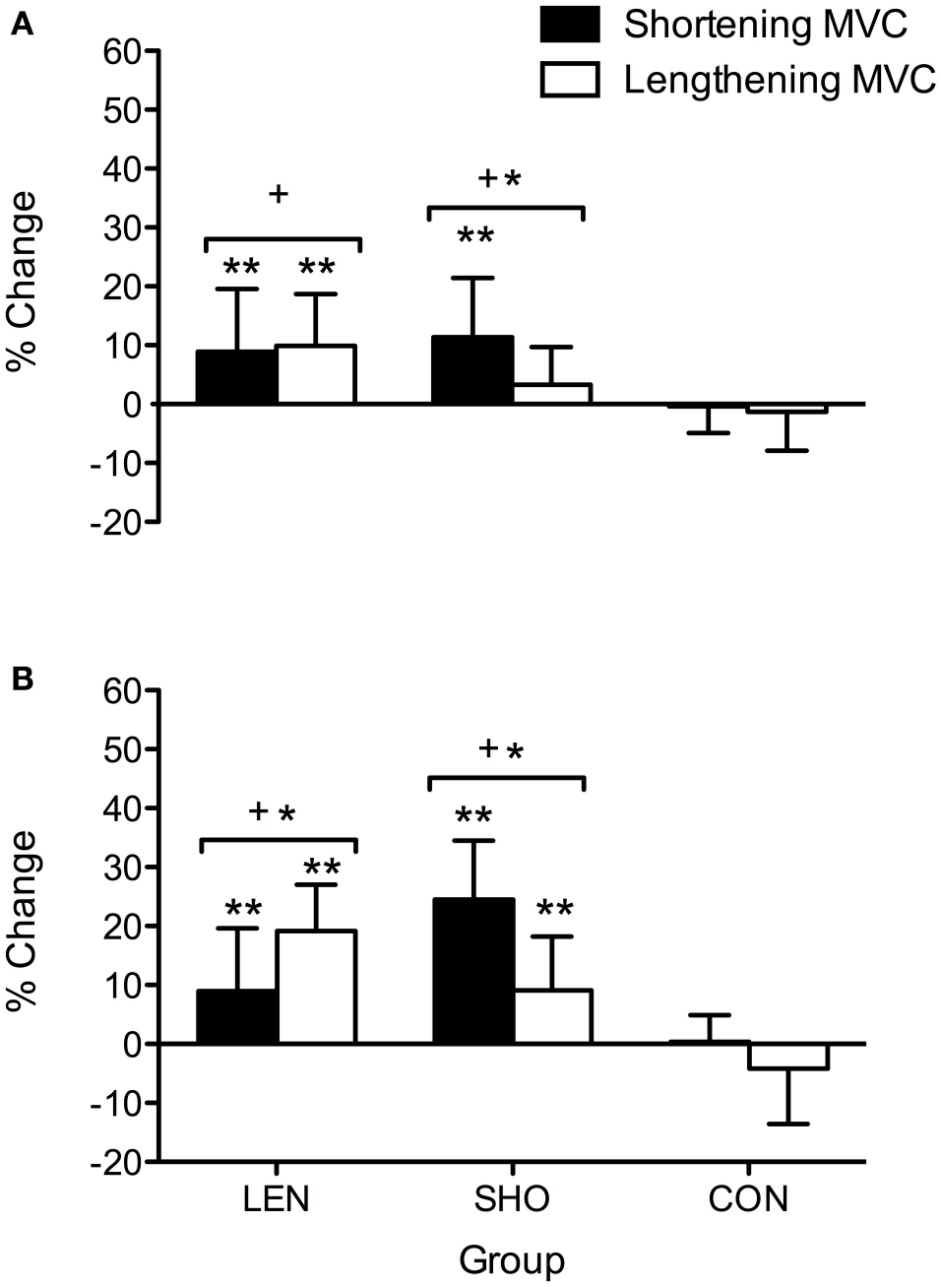
**Percentage change in shortening and lengthening MVC across time. (A)** Percentage change pre to mid. **(B)** Percentage change pre to post. ^*^Denotes significant difference between muscle contractions; ^+^significantly different to control group; ^**^Significantly different from pre-values.

The percentage increase in MVC pre to post training (Figure 1) showed a time effect [*F*_(2, 56)_ = 25.5; *P* < 0.001]. In the LEN group, *post-hoc* analysis showed MVC increased (*P* < 0.05) from pre to mid training for lengthening and shortening contractions. The SHO group showed an increase in MVC from pre to mid in shortening muscle contractions (*P* = 0.04; 95% CI 6.77–18.8%).

*Post-hoc* analysis also showed MVC increased from pre to post training for shortening and lengthening MVC in both the SHO (Shortening MVC increased 33.2–40.8 Nm; 95% CI 13.1–18.4%; *P* < 0.001: Lengthening MVC increased (51.8–56.4 Nm; 95% CI 2.60–15.8%; *P* = 0.006) and LEN (Shortening increase MVC; 33.8–38.5 Nm; 95% CI 3.30–14.6%; *P* = 0.009: Lengthening increased MVC; 49.5–58.6 Nm; 95% CI 13.7–24.6%; *P* < 0.001) training groups with no change in the CON group (*P* > 0.05).

A significant time-by-group-by-contraction type interaction was also seen [*F*_(4, 56)_ = 6.7; *P* < 0.001] for MVC; whereby the SHO group showed an increase in shortening MVC across time when compared to the lengthening MVC (*P* > 0.001). Similarly, the LEN group showed a greater increase in lengthening when compared to shortening MVC post training (19 vs. 9%; *P* = 0.02; CI = 3.1–17.4%). There was no significant difference in the CON group between the contraction types or across time.

### Corticospinal variables

**Figure 2:**
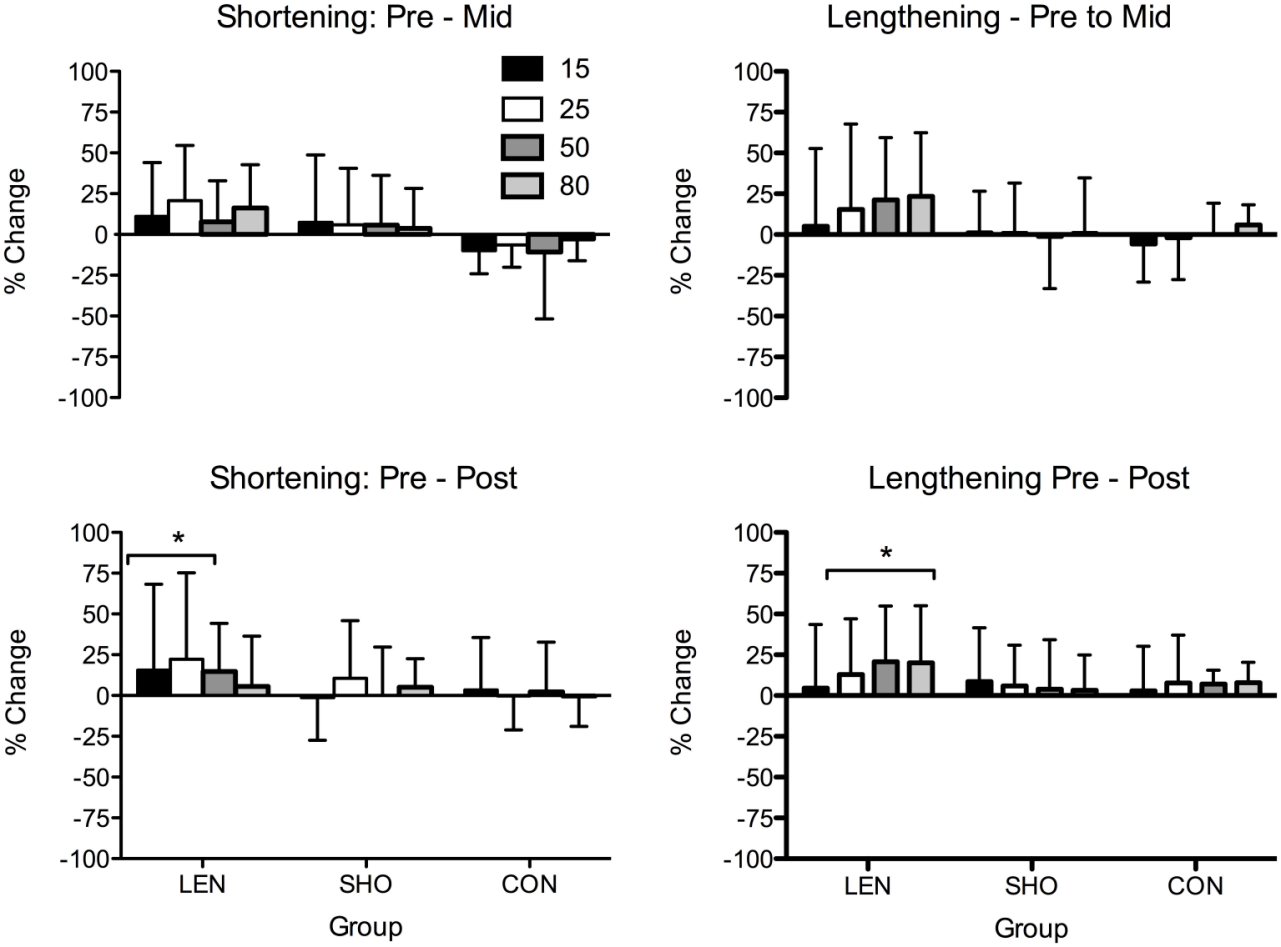
**Percentage change in shortening and lengthening MEP's in each group across time when expressed relative to M_MAX_**. ^*^Significantly different from pre-values.

**Figure 3:**
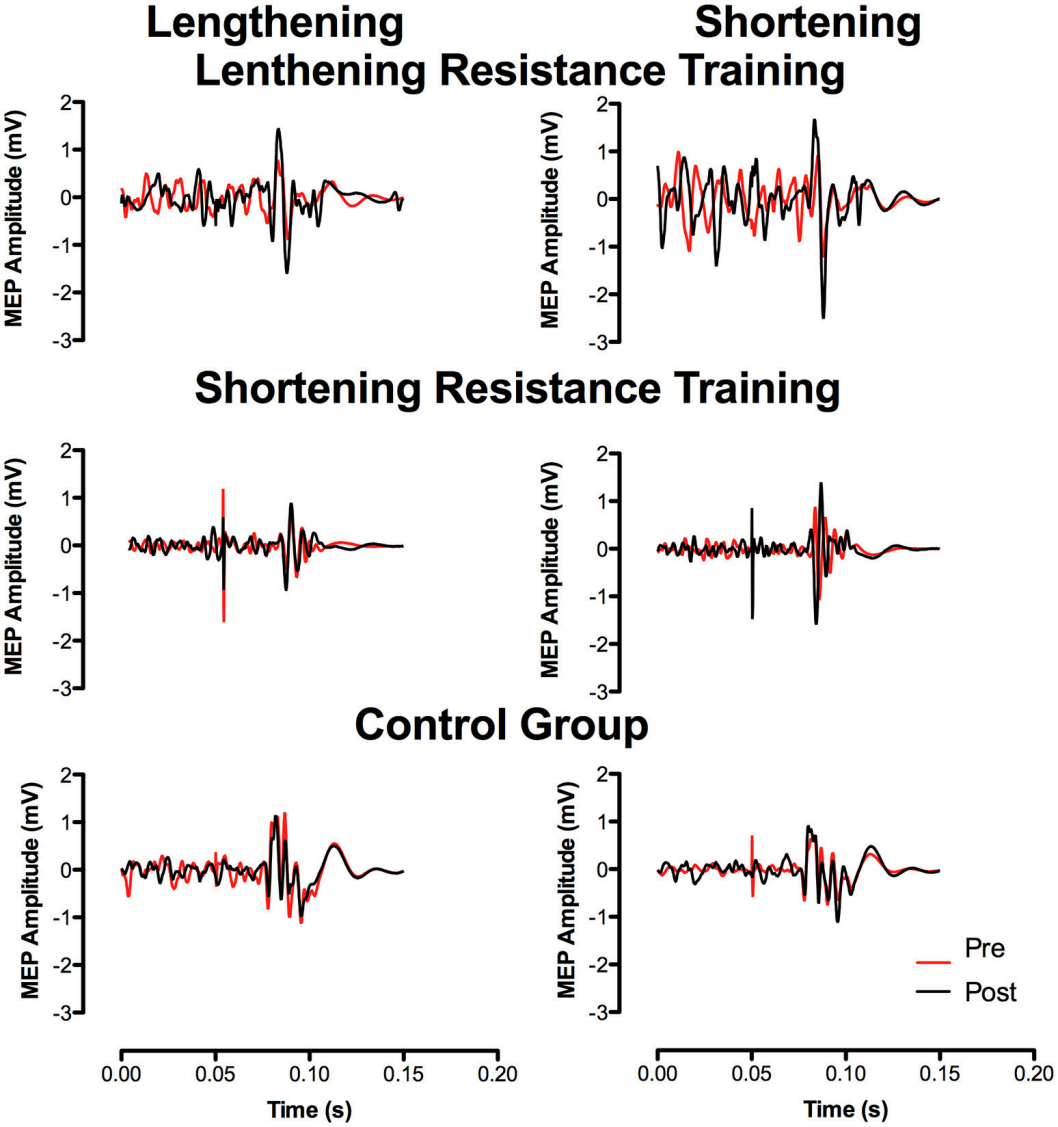
**Representative traces of MEP's pre and post resistance training recorded at 80% of relative MVC**.

No changes in resting MEP amplitude (Pre–Post: LEN = 1.3%, SHO = −9.4%, CON = −8.5%) or rMT were found across time (Pre–Post: LEN = 4.4%, SHO = 3.4%, CON = 0.7%). However, there was a main effect of time for MEP amplitude during an active muscle contraction [*F*_(2, 27)_ = 4.7; *P* = 0.01] when expressed relative to M_MAX_ (Figure 2). LEN group showed an increase in MEPs when relative to M_MAX_ during lengthening contractions. Differences were found at, 50% (*P* = 0.001; 95% CI 10.6–45.6%) and 80% (*P* < 0.001; 95% CI 10.3–46.2%) lengthening contraction intensity (Figure 2). There were no difference between contraction type and groups (*P* > 0.05). A representative trace of MEPs pre-post training can be seen in (Figure 3).

**Figure 4:**
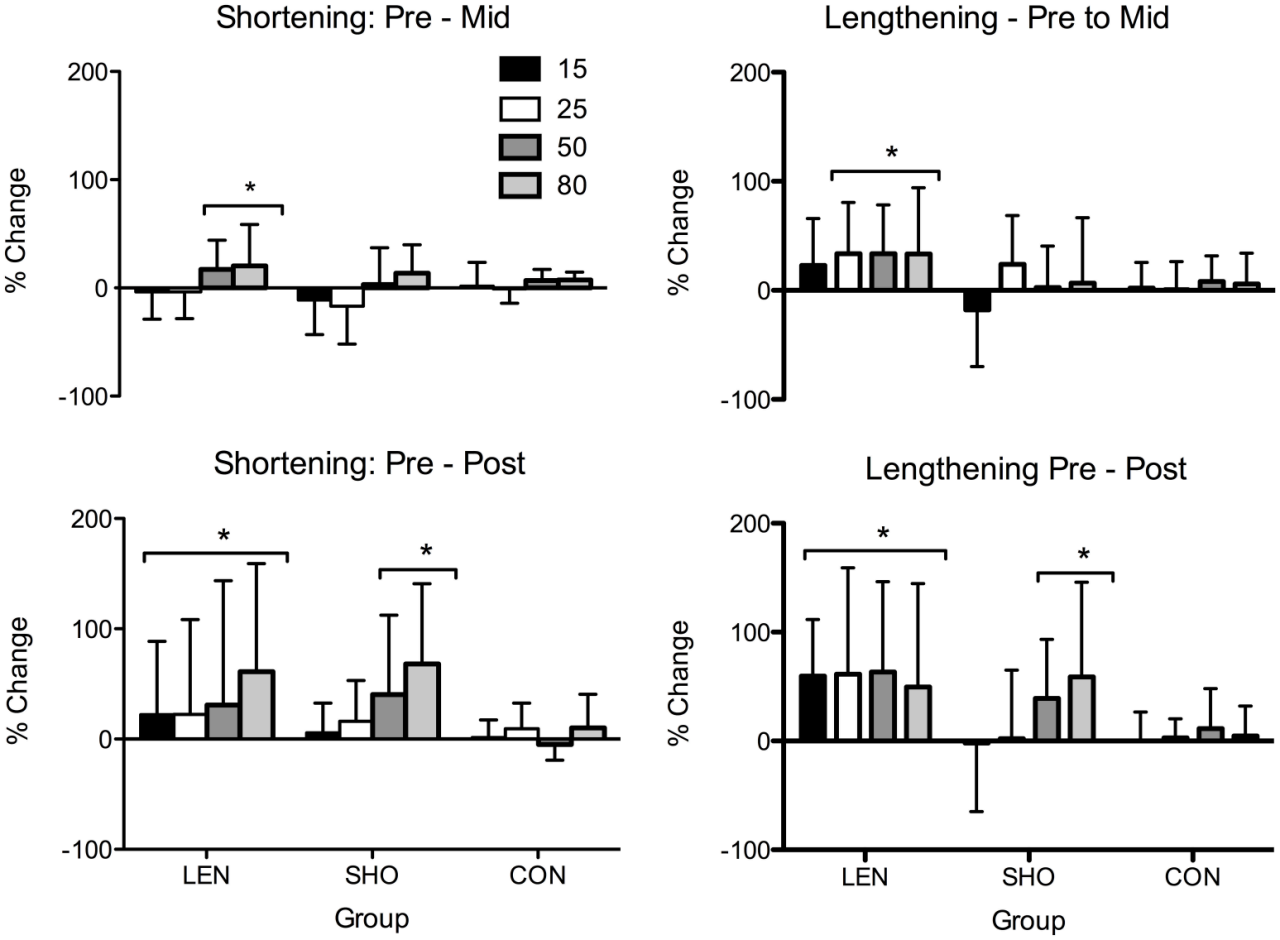
**Percentage change in shortening and lengthening MEP's in each group across time when expressed relative to M_MAX_ and background EMG**. ^*^Significantly different from pre-values.

MEPs expressed relative to background EMG (Figure 4) revealed a main effect across time [*F*_(2, 27)_ = 18.4; *P* < 0.001]. Significant increases were only seen in the LEN (Figure 4). For MEP's relative to background EMG and M_MAX_ significant increases were seen across multiple contraction intensities and both contraction types in the LEN group with no (*P* > 0.05) changes in the control group. The SHO group showed an increase at intensities 50 and 80% of shortening and lengthening MVC. There was also a group × time interaction [*F*_(4, 56)_ = 4.1; *P* = 0.006] demonstrating both experimental groups increased compared to the control and baseline. However there was no significant difference between groups (*P* > 0.05). No differences were found between the contraction type and groups (*P* > 0.05). Additionally, there was no significant change in the corticospinal silent period across time (*P* > 0.05).

### PNS

There was no change (*P* > 0.05) in M_MAX_ across the study. H-reflex showed no difference (*P* > 0.05) across time or between groups when expressed relative to M_MAX_ and background EMG.

#### V-wave pre to post

**Figure 5:**
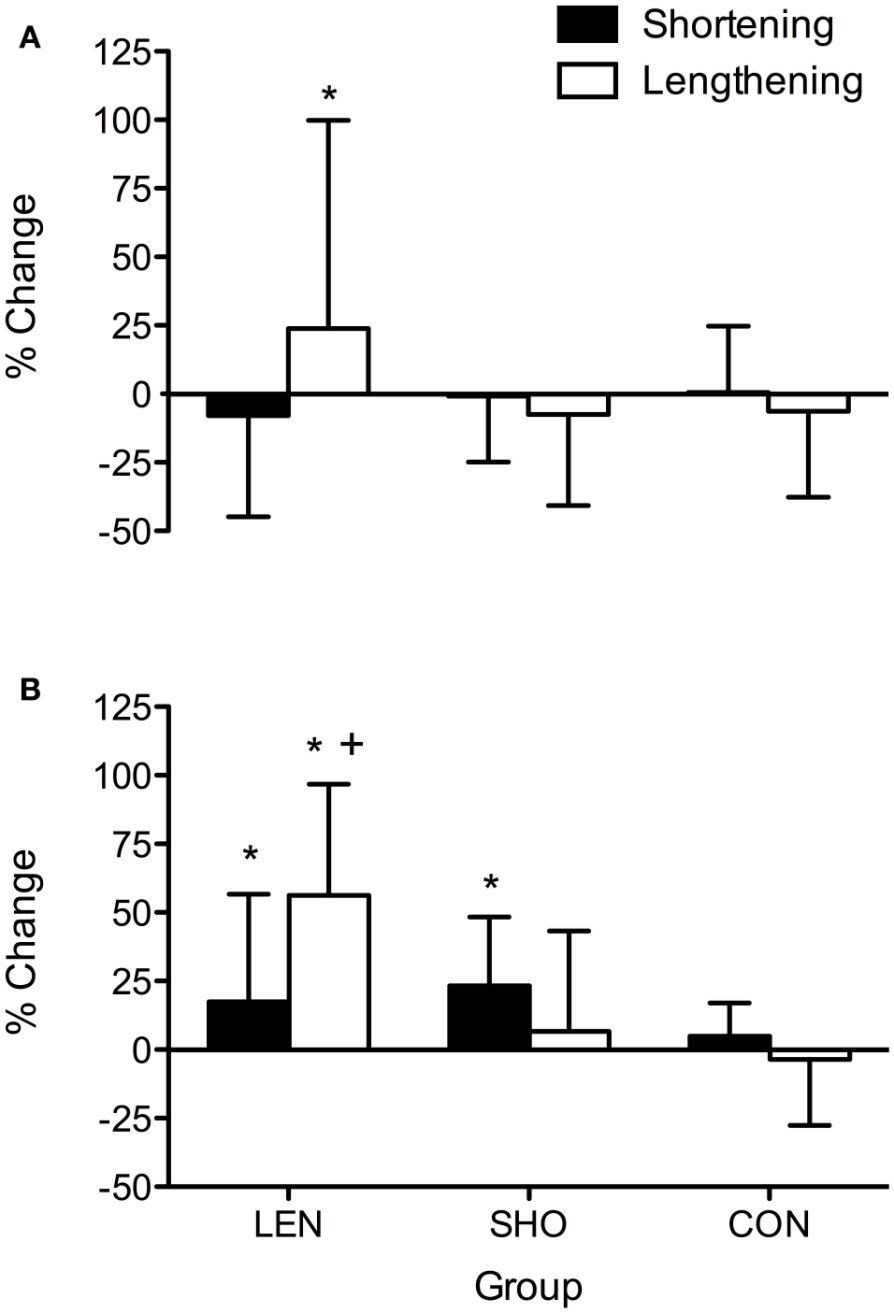
**Percentage change in shortening and lengthening V-wave amplitude relative to M_MAX_ across time (A)** Percentage change pre to mid. **(B)** Percentage change pre to post. ^*^Denotes significant difference from pre-values; ^+^Significantly different from SHO and CON group.

An increase in V-wave amplitude (Figure 5) across time was observed [*F*_(2, 52)_ = 6.0; *P* = 0.004]. Pairwise comparisons showed the LEN groups V-wave significantly increased during lengthening MVC's (0.29 ± 0.12–0.46 ± 0.15 V-wave/M_MAX_; *P* < 0.001; CI = 44.5–88.9%) and during shortening MVC's 0.45 ± 0.17–0.53 ± 0.20 V-wave/M_MAX_; *P* = 0.02; CI = 7.3–45.3%). The SHO group, there was only an increase in V-wave amplitude during shortening MVC's 0.43 ± 0.18–0.53 ± 0.19 V-wave/M_MAX_; *P* = 0.03; CI = 7.3–45.3%). No change (*P* > 0.05) was found in the CON group. Additionally, there was a group-by-contraction interaction [*F*_(2, 26)_ = 8.0; *P* = 0.002]. LEN group V-wave showed a greater increase compared to the shortening groups V-wave during shortening contractions (*P* = 0.003; CI = 29.3–89.4%; Figure 5).

#### Detraining

##### Maximal voluntary contraction

**Figure 6:**
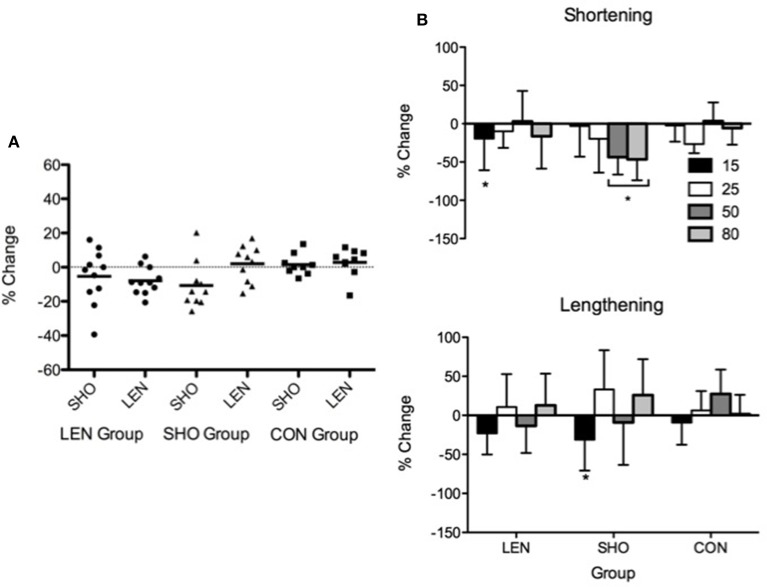
**Individual and mean percentage change in shortening and lengthening MVC following 2 weeks detraining**. Solid line represents the mean response and the symbols represent individual changes **(A)**. Percentage change in shortening and lengthening MEP's relative to M_MAX_ and background EMG following 2 weeks detraining. ^*^Denotes significant difference from pre-values **(B)**.

There were no differences (*P* > 0.05) in SHO and LEN MVC (Figure 6) following 2 weeks of detraining in all groups.

### Corticospinal

rMT, resting MEPs, and MEPs during an active muscle contraction did not change following 2 weeks detraining (*P* > 0.05). However, relative to background EMG there was a significant decrease [*F*_(1, 27)_ = 10.2; *P* < 0.01] in MEPs (Figure 6). There was a significant interaction [*F*_(5, 83)_ = 3.1; *P* = 0.01], the SHO group showed a significant decrease during 50% (−44%; *P* < 0.001; CI = 19.4–59.1%), 80% (−47%; *P* = 0.001; CI = 18.3–61.8%) shortening MVC and 15% lengthening MVC (−31%; *P* = 0.01; CI = 7.7–45.8%). There was no change in the corticospinal silent period.

### Peripheral neuromuscular stimulation

H-reflex or V-wave amplitude (*P* > 0.05) did not significantly change after the detraining period.

## Discussion

### Resistance training

The main findings from the first part of this study were: (1) shortening resistance training improved shortening MVC more than lengthening MVC, and lengthening resistance training improved lengthening MVC more than shortening MVC, respectively; (2) corticospinal excitability increase for 50 and 80% lengthening contraction in the LEN group, however for a relative motorneuron pool (background EMG), corticospinal excitability increased for both contraction types in both groups, and (3) V-wave increased when tested during shortening and lengthening muscle contractions in the LEN group, but only increased during shortening muscle contractions in the SHO group, showing evidence of task specificity. Additionally, for the first time we have demonstrated lengthening resistance training increases V-wave amplitude during the lengthening test contraction to a greater extent than shortening resistance training and increased V-wave amplitude during shortening test contraction.

In line with previous research, a recent meta analysis showed (Roig et al., 2009), task specific improvements in MVC. More specifically, lengthening training increased lengthening strength more than shortening training and vice-versa. Notwithstanding, the current study increases our understanding of the neurological adaptations arising from resistance training at a corticospinal and spinal level following 4 weeks of resistance training. These data support previous work demonstrating little change in corticospinal excitability at rest (Carroll et al., 2002, 2009); however, during an active lengthening contraction in the LEN group, there was an increase in corticospinal excitability when expressed relative to M_MAX_. The reason for this observation is probably linked to the differences in corticospinal and spinal balance of excitatory and inhibitory circuits during lengthening muscle contractions. Hortobágyi et al. (1996) demonstrated a seven times greater increase in lengthening background EMG from lengthening compared to shortening resistance training. The increases in background EMG were attributed to a greater recruitment of type II fibers during lengthening contractions (Hortobágyi et al., 1996), though this is unlikely to explain amplitude changes in MEP's. The superiority of lengthening resistance training has previous been thought to be due to the higher absolute loads that are completed during training (Roig et al., 2009); although it has also been shown that when lengthening training was matched to the same absolute loads as shortening resistance training there were no differences in contractions specific MVC. Consequently, it appears that lengthening contractions are a more powerful stimulus to modulate changes in cortiospinal excitability.

Contrary to previous results (Carroll et al., 2002), these data demonstrate that when MEPs were expressed relative to background EMG, there was a significant increase in MEP peak-to-peak amplitude from pre to post resistance training. Expressing MEP's relative to background EMG, represents the excitability of corticospinal cells for a particular level of muscle activity (Carroll et al., 2002). Representing MEP's to background EMG takes into consideration modifications in muscle activity through changes in strength. Carroll et al. (2002) suggested that fewer motorneurons were activated relative to background EMG due to a change in firing rates and/or intrinsic properties of motorneurons. We propose that for a similar or lower level of background EMG, the cortiospinal pathway is more excitable and consequently the threshold of corticospinal neurons is lower for a given force. The type of resistance training protocols used in each study could explain differences in the findings. For example, performing precision tasks that require an element of complexity or skill can increase corticospinal excitability (Perez et al., 2004). Carroll et al. (2002) participants' were required to isometrically resist an external force, the skill element of this arguably, is considerably less. Even though our results show corticospinal excitability increased following strength training, complexity of the movement appears to play a significant role with changes in corticospinal excitability. Net output from motoneurons projecting to the trained muscle has been shown to increase following a single resistance training session (Nuzzo et al., 2016). Previous data from our laboratory (Tallent et al., 2012) has also shown MEP's to increase following a single isokinetic training session. This further indicates that corticospinal excitability may have only a limited role in strength training, and is more associated with the acquiring the motor patterns of movement. Further research should focus on a greater number of assessment time points in the first 4 weeks of resistance training to increase our understanding of the role of corticospinal excitability in skill and resistance training.

Maximal lengthening resistance training has been suggested to cause a decrease in inhibition associated with performing lengthening muscle contractions (Aagaard, 2003). This study found no evidence of modifications of the silent period following the training period in any group, suggesting corticospinal inhibition, as measured here, was not responsible for modulating the MEP peak-to-peak amplitude and strength improvement. Previous resistance training studies have shown both a decrease (Kidgell and Pearce, 2010; Latella et al., 2012) and no change in corticospinal inhibition (Kidgell et al., 2010). Recent work using paired-pulse TMS has shown lower short-interval intracortical inhibition to occur after a period of resistance training (Weier et al., 2012). Future research should include paired pulse TMS paradigms to further understand any modulation in inhibitory and facilitation networks that influence corticospinal excitability following resistance training.

The increases in V-waves found in this study suggest that an increase in efferent output supports the increase in strength early in a resistance training programme. The lack of changes in inhibition suggest that the greater efferent cortical output is needed to overcome the cortiospinal inhibition and increase the motor unit activation. Changes in V-wave amplitude seem to reveal a consistent pattern across different studies and training paradigms (Duclay et al., 2008; Ekblom, 2010; Vila-Chã et al., 2012) with increases of up to 80% (Gondin et al., 2006). Our findings further support this by showing increased V-wave in both training groups; however, for the first time we have shown a greater response to lengthening resistance training. A meta-analysis (Roig et al., 2009) showed greater gains in total strength (lengthening and shortening MVC combined), which may offer some explanation that an enhancement in efferent output during MVC, at least in part, might be responsible for the greater increases in strength associated with lengthening resistance training. Even though lengthening contractions are suggested to have a greater supraspinal output (Fang et al., 2001, 2004) and greater inhibition at a spinal level (Duclay and Martin, 2005; Duclay et al., 2008), we hypothesized that the greater CNS adaptations are due to a further increase in supraspinal output. However, tension regulating mechanisms (such as the golgi tendon organ) cannot be excluded as adaptations for supporting the increase in strength. However, it should be noted that the starting value of lengthening V-wave in the LEN group was visually lower compared to the other groups, reasons for this are unclear.

Previous research suggests a lack of change in resting H-reflex (Aagaard et al., 2002; Holtermann et al., 2007; Duclay et al., 2008; Ekblom, 2010), however, during an active contraction, there are reports of increased spinal excitability (Holtermann et al., 2007; Duclay et al., 2008). This is likely attributable to a reduction in presynaptic inhibition (Aagaard et al., 2002). Given our data found no changes in H-reflex, our laboratory supports previous work suggesting changes in strength have tenuous links with the Ia afferent loop, We examined H-reflex during a relatively low intensity contraction (~25% MVC) in an attempt to detect changes at a spinal level, but did not observe any change. It is possible that any spinal adaptation may occur at higher contraction intensities that are specific to training intensity and we acknowledge this as a limitation.

### Detraining

The second part of this study revealed that maximal strength and V-wave amplitude were both retained following a 2 week detraining period independent of the contraction type used during the training period and corticospinal excitability decreased following 2 weeks detraining. Studies have shown an increase in strength above baseline from weeks to months following cessation of resistance training in healthy and special population such as the elderly (Carvalho et al., 2009; Popadic Gacesa et al., 2011; Correa et al., 2013). A recent meta-analysis concluded that following the cessation of training, a decrease in maximal force was evident in the third week of inactivity (Bosquet et al., 2013). However, given the relatively short duration of our training programme and the notion that longer resistance training programmes lead to longer-lasting adaptations (Bosquet et al., 2013), it is perhaps surprising that in the current study, detraining did not cause a reduction in maximal voluntary force. With so many diverse resistance training paradigms and different periods no activity, little definite conclusions can be made from the literature. Despite this, our results found evidence of neurological changes following 2 weeks post the cessation of resistance training.

Both groups showed a decrease from post resistance training MEP amplitude when expressed relative to background EMG. There are few studies that have investigated detraining at a cortical or spinal level. Although statistical analyses (*n* = 4) were not performed, Jensen et al. (*2005*) appeared to show a reduction in the MEP recruitment curve toward baseline values following cessation of resistance training in the biceps brachii muscle. Whether this is due to a withdrawal of the resistance training stimulus or a reduction in strength is unclear. Previous research has showed conflicting results following periods of inactivity (with no prior resistance training) in rodents and humans; an increase in corticospinal excitability (Roberts et al., 2007), reduced cortical representation/excitability (Leukel et al., 2014) or no change has been reported (Zanette et al., 1997). The lack of research in this area leaves definitive conclusions difficult. Others have suggested (Clark et al., 2006) that neurological factors contribute to ~48% of the loss in strength from inactivity. Our results add to this body of evidence and show that corticospinal excitability rapidly returns toward baseline following the cessation of strength training. Our results add to Jensen et al.'s (2005) work by demonstrating that the reduction in corticospinal excitability may largely be due to the cessation of the resistance training.

Long periods of inactivity have been shown to decrease spinal excitability (Yamanaka et al., 1999). Given there was no change in spinal excitability from the resistance-training programme it is unsurprising there was no change following 2 weeks of detraining. Unlike voluntary activation (Gondin et al., 2006), which seems to decrease following a period of inactivity, V-wave was maintained following 2 weeks detraining. However, given that there was only 2 weeks detraining it is difficult, to conclude that V-wave is a longer lasting neurological adaptation; nonetheless it does not see reductions in this epoch. Furthermore, these data cannot rule out the notion that the maintenance of MVC may in part be attributable to a preservation of neural drive, and supports the hypothesis that volitional drive is strongly associated with strength loss.

Despite this study being the first study to investigate site specific adaptations from shortening and lengthening resistance training, the muscle used during the resistance training could be considered a limitation given its applicability to other muscles that might be targetted during resistance training programs. Further research should focus on changes in corticospinal and spinal excitability the agonist antagonist relationship. In addition, an extended period of detraining to observe strength loss would allow for more definite conclusions on the time course and underlying mechanisms behind detraining-related losses in strength.

In conclusion, this study showed task-specific gains in strength that were greater following lengthening training. The increase in maximal muscle force generating capacity was accompanied with changes in corticopsinal excitability and volitional drive. These V-wave changes were also contraction specific and were greatest from lengthening resistance training, suggesting that efferent neural output at a cortical level increases significantly more from lengthening resistance training. Even though MVC was maintained following 2 weeks of detraining, there was a significant reduction in corticospinal excitability (relative to background EMG). As a decrease in corticospinal excitability occurred from lengthening and shortening resistance training, this study showed little evidence of the notion that lengthening action causes longer lasting neurological adaptations following short term resistance training.

## Author contributions

JT, SG, KG, TH, and GH conceived and planned the study. JT, SG, KG, and GH performed the study. JT, SG, KG, TH, and GH analyzed the data and JT, SG, and GH ran statistical analysis. JT wrote the first draft with contributes from SG, KG, TH, and GH. All authors approved the final draft.

### Conflict of interest statement

The authors declare that the research was conducted in the absence of any commercial or financial relationships that could be construed as a potential conflict of interest.
