# Supplementary material for: Enhanced Corticospinal Excitability and Volitional Drive in Response to Shortening and Lengthening Strength Training and Changes Following Detraining
Source: Front Physiol. 2017 Feb 7;8:57. doi: 10.3389/fphys.2017.00057 (PMC5293799; doi:10.3389/fphys.2017.00057)
Supplement: Supplementary file 1 [file DataSheet1.docx]

**Supplementary Table 1.** MEP’s relative to M_MAX_, across time, contraction intensity, groups and during shortening and lengthening contractions.

|  | | Shortening | | | | | | | | | | | | | |  |  | | | |  |  |  |
| --- | --- | --- | --- | --- | --- | --- | --- | --- | --- | --- | --- | --- | --- | --- | --- | --- | --- | --- | --- | --- | --- | --- | --- |
|  | | Pre | | | | | Mid | | | | | Post | | | | Detraining | | | | | | | |
|  | 15% | | 25% | 50% | 80% | 15% | | 25% | 50% | 80% | 15% | | 25% | 50% | 80% | 15% | | 25% | 50% | 80% | | | |
| LEN | 0.34±0.11 | | 0.38±0.14 | 0.49±0.16 | 0.58±0.21 | 0.38±0.11 | | 0.46±0.12 | 0.52±0.11 | 0.67±0.16 | 0.39±0.16 | | 0.46±0.19 | 0.56±0.16 | 0.61±0.19 | 0.38±0.14 | | 0.40±0.16 | 0.49±0.17 | 0.57±0.20 | | | |
| SHO | 0.34±0.12 | | 0.36±0.11 | 0.44±0.13 | 0.48±0.09 | 0.36±0.12 | | 0.39±0.12 | 0.47±0.17 | 0.50±0.17 | 0.33±0.13 | | 0.40±0.13 | 0.44±0.14 | 0.50±0.15 | 0.38±0.11 | | 0.33±0.14 | 0.42±0.11 | 0.45±0.11 | | | |
| CON | 0.33±0.10 | | 0.34±0.11 | 0.44±0.12 | 0.49±0.12 | 0.30±0.11 | | 0.34±0.14 | 0.39±0.17 | 0.48±0.14 | 0.34±0.09 | | 0.36±0.09 | 0.45±0.10 | 0.49±0.10 | 0.34±0.12 | | 0.37±0.12 | 0.45±0.14 | 0.44±0.19 | | | |
|  | | Lengthening | | | | | | | | | | | | | | | | | | | | | |
| LEN | 0.31±0.13 | | 0.33±0.15 | 0.41±0.18 | 0.49±0.24 | 0.32±0.11 | | 0.39±0.14 | 0.50±0.20 | 0.51±0.12 | 0.32±0.13 | | 0.38±0.13 | 0.50±0.20***** | 0.59±0.18* | 0.31±0.14 | | 0.35±0.13 | 0.42±0.15 | 0.55±0.20 | | | |
| SHO | 0.27±0.10 | | 0.31±0.08 | 0.41±0.08 | 0.49±0.11 | 0.28±0.10 | | 0.31±0.09 | 0.41±0.12 | 0.50±0.14 | 0.30±0.10 | | 0.33±0.08 | 0.43±0.07 | 0.51±0.14 | 0.25±0.07 | | 0.30±0.10 | 0.37±0.12 | 0.46±0.11 | | | |
| 3CON | 0.31±0.10 | | 0.32±0.08 | 0.40±0.09 | 0.48±0.14 | 0.29±0.10 | | 0.31±0.10 | 0.40±0.11 | 0.45±0.11 | 0.30±0.09 | | 0.34±0.10 | 0.43±0.08 | 0.49±0.14 | 0.28±0.11 | | 0.33±0.12 | 0.42±0.13 | 0.49±0.15 | | | |

* Significantly different from pre values; LEN lengthening, SHO shortening, CON Control.

**Supplementary Table 2.** V-wave relative to M_MAX_, across time, groups and during shortening and lengthening contractions.

|  | Shortening | | | | Lengthening | | | |
| --- | --- | --- | --- | --- | --- | --- | --- | --- |
|  | Pre | Mid | Post | Detraining | Pre | Mid | Post | Detraining |
| ECC | 0.45±0.17 | 0.41±0.16 | 0.53±0.20* | 0.46±0.17 | 0.29±0.12 | 0.36±0.15 | 0.46±0.15* ^†^ | 0.39±0.17 |
| SHO | 0.43±0.18 | 0.43±0.17 | 0.53±0.19* | 0.43±0.23 | 0.44±0.17 | 0.40±0.20 | 0.47±0.20 | 0.35±0.21 |
| CON | 0.42±0.14 | 0.42±0.14 | 0.44±0.13 | 0.44±0.15 | 0.39±0.13 | 0.36±0.12 | 0.37±0.14 | 0.38±0.19 |

* Significantly different from pre values, ^†^ Significantly different SHO lengthening post; LEN lengthening, SHO shortening, CON Control.

**Supplementary Table 3.** H-reflex relative to M_MAX_ at 25% MVC , across time, groups and during shortening and lengthening contractions.

|  | Shortening | | | Lengthening | | |  |  |
| --- | --- | --- | --- | --- | --- | --- | --- | --- |
|  | Pre | Mid | Post | Detraining | Pre | Mid | Post | Detraining |
| ECC | 0.14±0.04 | 0.14±0.05 | 0.14±0.03 | 0.13±0.04 | 0.10±0.05 | 0.10±0.02 | 0.10±0.02 | 0.10±0.04 |
| SHO | 0.15±0.07 | 0.17±0.08 | 0.13±0.05 | 0.13±0.04 | 0.13±0.07 | 0.13±0.05 | 0.13±0.07 | 0.14±0.05 |
| CON | 0.11±0.04 | 0.11±0.04 | 0.12±0.04 | 0.13±0.03 | 0.09±0.04 | 0.10±0.03 | 0.10±0.03 | 0.10±0.04 |

LEN lengthening, SHO shortening, CON Control, MVC maximal voluntary contractions.
